# Deep learning can predict survival directly from histology in clear cell renal cell carcinoma

**DOI:** 10.1371/journal.pone.0272656

**Published:** 2022-08-17

**Authors:** Frederik Wessels, Max Schmitt, Eva Krieghoff-Henning, Jakob N. Kather, Malin Nientiedt, Maximilian C. Kriegmair, Thomas S. Worst, Manuel Neuberger, Matthias Steeg, Zoran V. Popovic, Timo Gaiser, Christof von Kalle, Jochen S. Utikal, Stefan Fröhling, Maurice S. Michel, Philipp Nuhn, Titus J. Brinker

**Affiliations:** 1 Digital Biomarkers for Oncology Group, National Center for Tumor Diseases (NCT), German Cancer Research Center (DKFZ), Heidelberg, Germany; 2 Department of Urology & Urological Surgery, Medical Faculty Mannheim of Heidelberg University, University Medical Center Mannheim, Mannheim, Germany; 3 Department of Medicine III, University Hospital RWTH Aachen, Aachen, Germany; 4 Division of Pathology and Data Analytics, Leeds Institute of Medical Research at St James’s, University of Leeds, Leeds, United Kingdom; 5 Medical Oncology, National Center for Tumor Diseases (NCT), University Hospital Heidelberg, Heidelberg, Germany; 6 Institute of Pathology, Medical Faculty Mannheim of Heidelberg University, University Medical Center Mannheim, Mannheim, Germany; 7 Department of Clinical-Translational Sciences, Berlin Institute of Health (BIH), Charité University Medicine, Berlin, Germany; 8 Clinical Cooperation Unit Dermato-Oncology, University Medical Center Mannheim, University of Heidelberg, German Cancer Research Center (DKFZ), Mannheim and Heidelberg, Germany; 9 National Center for Tumor Diseases, German Cancer Research Center (DKFZ), Heidelberg, Germany; Wroclaw University of Science and Technology, POLAND

## Abstract

For clear cell renal cell carcinoma (ccRCC) risk-dependent diagnostic and therapeutic algorithms are routinely implemented in clinical practice. Artificial intelligence-based image analysis has the potential to improve outcome prediction and thereby risk stratification. Thus, we investigated whether a convolutional neural network (CNN) can extract relevant image features from a representative hematoxylin and eosin-stained slide to predict 5-year overall survival (5y-OS) in ccRCC. The CNN was trained to predict 5y-OS in a binary manner using slides from TCGA and validated using an independent in-house cohort. Multivariable logistic regression was used to combine of the CNNs prediction and clinicopathological parameters. A mean balanced accuracy of 72.0% (standard deviation [SD] = 7.9%), sensitivity of 72.4% (SD = 10.6%), specificity of 71.7% (SD = 11.9%) and area under receiver operating characteristics curve (AUROC) of 0.75 (SD = 0.07) was achieved on the TCGA training set (n = 254 patients / WSIs) using 10-fold cross-validation. On the external validation cohort (n = 99 patients / WSIs), mean accuracy, sensitivity, specificity and AUROC were 65.5% (95%-confidence interval [CI]: 62.9–68.1%), 86.2% (95%-CI: 81.8–90.5%), 44.9% (95%-CI: 40.2–49.6%), and 0.70 (95%-CI: 0.69–0.71). A multivariable model including age, tumor stage and metastasis yielded an AUROC of 0.75 on the TCGA cohort. The inclusion of the CNN-based classification (Odds ratio = 4.86, 95%-CI: 2.70–8.75, p < 0.01) raised the AUROC to 0.81. On the validation cohort, both models showed an AUROC of 0.88. In univariable Cox regression, the CNN showed a hazard ratio of 3.69 (95%-CI: 2.60–5.23, p < 0.01) on TCGA and 2.13 (95%-CI: 0.92–4.94, p = 0.08) on external validation. The results demonstrate that the CNN’s image-based prediction of survival is promising and thus this widely applicable technique should be further investigated with the aim of improving existing risk stratification in ccRCC.

## Introduction

Renal cell carcinoma (RCC) belongs to the fifteen most common cancers worldwide [1]. Surgical removal of the tumor is recommended for localized stages and oligometastatic cases while non-resectable metastases require systemic therapy and facultative cytoreductive nephrectomy [2]. In the existing diagnostic and therapeutic algorithms, risk stratification plays an essential role. For example, after curative surgery, risk-adapted follow-up is recommended [2]. Histological subtype, high tumor grade, more advanced tumor stage and others are possible risk factors of early recurrence in RCC and are thus included in current risk models [3, 4].

The use of artificial intelligence (AI) has shown impressive results across many different medical fields in medicine [5–9]. In image analysis of histological slides, AI has recently gained momentum with promising results across different tumor entities. For instance, high accuracy was reported for automated tumor detection and grading in genitourinary cancers [10]. Moreover, studies demonstrated that AI was able to detect mutations based on hematoxylin and eosin-stained (H&E) slides in a variety of tumor types [11, 12] including genitourinary tumors [13, 14]. These mutations apparently lead to morphological changes which are detectable by artificial intelligence techniques, especially convolutional neural networks (CNNs). Such works have laid the ground for the rising number of studies investigating AI for the prediction of oncological outcomes [15, 16].

Survival prognosis as determined by current risk calculators, such as the IMDC risk calculator, is used to guide therapy decisions [2, 17, 18]. So far, no models based on artificial intelligence are currently used in clinical practice. However, first studies were conducted to explore the potential of such models in the prediction of survival [19–23]. In one study, a CNN was able to stratify risk into a high- and a low-risk group in patients with stage I clear cell RCC (ccRCC) using H&E slides [23]. Similar results were obtained in another study, where tumor and nuclei features were extracted from H&E slides, which also allowed a significant risk stratification in terms of survival [21]. These positive results, however, were achieved using the Kidney Renal Clear Cell Carcinoma (KIRC) cohort from The Cancer Genome Atlas (TCGA) only. While this dataset certainly shows a degree of heterogeneity, the same cohort was used for training and testing of the respective models, so that overfitting cannot be excluded. To demonstrate generalizability to data from another source, validation of such methods on an external, independent dataset is necessary. We therefore developed a CNN algorithm that uses routine H&E slides for the prediction of overall survival in ccRCC and validate this algorithm externally. Furthermore, we compared its performance with that of a model based on known clinicopathological risk factors and generated a combined model. Our main goal was to contribute to further improving survival risk stratification in renal cell carcinoma in the future.

## Materials and methods

### Study population

The design and reporting of this work was done on the basis of the TRIPOD checklist [24]. The TCGA-KIRC cohort was screened for eligible patients and slides. The following inclusion criteria had to be fulfilled:

- Histologically confirmed diagnosis of ccRCC
- Availability of a diagnostic H&E-stained slide of the primary carcinoma used for routine diagnosis
- Follow-up information ≥ 60 months (5y-OS(**+**)) or death within 60 (5y-OS(**-**)) months after diagnosis

Patients / H&E slides were excluded for the following reasons:

- H&E slide containing <250 patches of ccRCC tissue of sufficient quality
- Follow-up of < 60 months for patients with last known vital status “alive”

For the independent validation cohort, patients with histologically confirmed ccRCC from the University Medical Center Mannheim who had undergone surgery between 2009 and 2011 were selected (in-house validation cohort). The analysis was approved by the local ethics committee (2015-849N-MA). Patients had given informed written consent to tissue analysis.

### Study design

The basic principle in using a CNN for image analysis is always similar. First, the image must be divided into smaller patches, which represent the input for the CNN. Then the CNN is trained using a training set. Here, the CNN is fed with the patches and the associated label that is to be classified or predicted. In this process, the CNN should learn which features of the image are relevant for the prediction of the label. In the case of binary prediction, the output of the CNN is usually a probability that can be converted into a binary prediction by means of a cut-off value (e.g. 0.5). After the training is completed, images from a validation set are fed to the CNN without the label being given to the CNN. After the prediction by the CNN, the predicted and true labels are compared and evaluated using different performance metrics.

**Fig 1:**
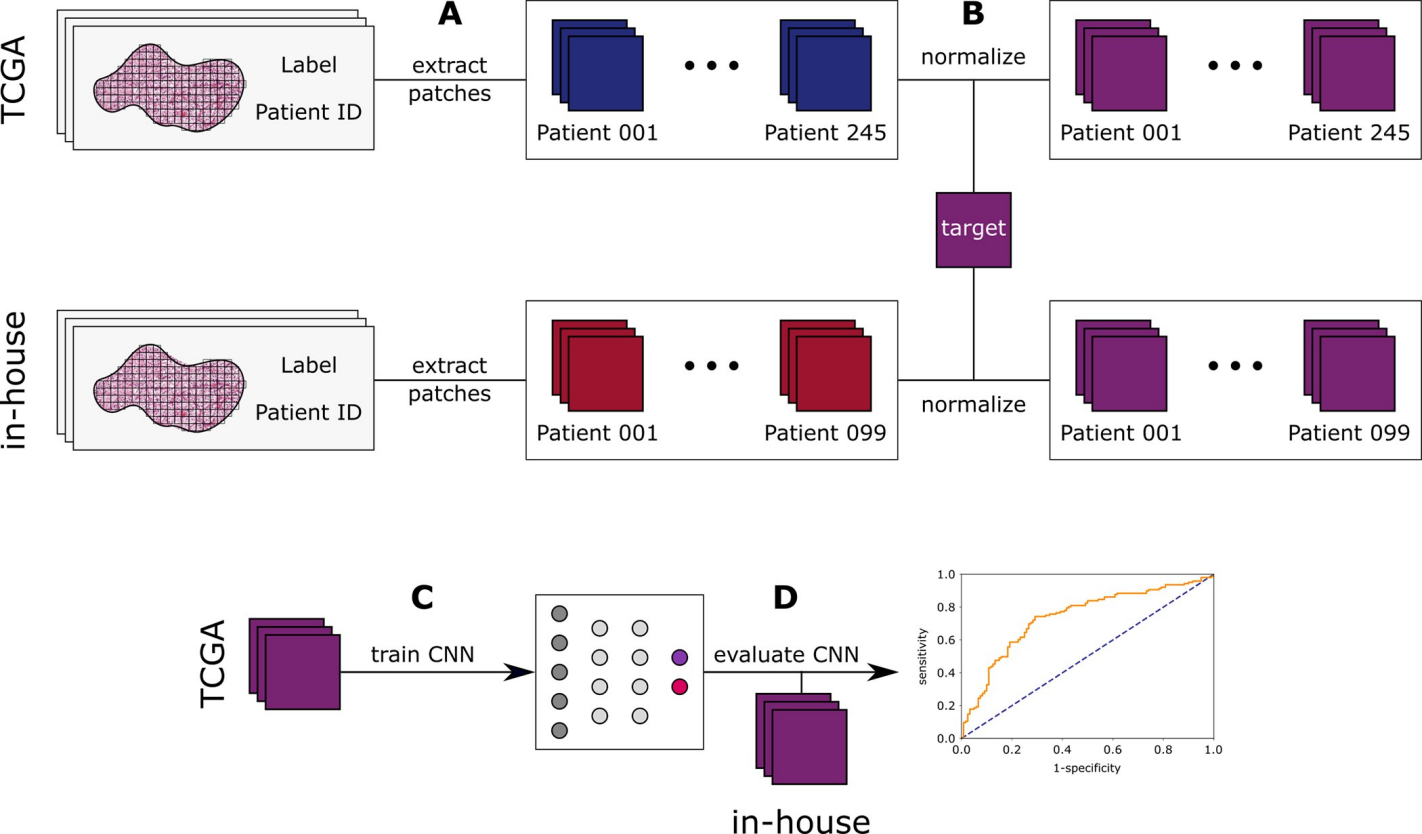
Preprocessing and workflow. (A) All annotated whole slide images from TCGA (training set) and from the validation cohort from our institution (independent test set) were tessellated into patches and downscaled as appropriate. Blurry patches were discarded. All patches were saved with a reference to their original WSI. TCGA = The Cancer Genome Atlas. (B) All patches were normalized with the same target, using the method as described by Macenko et al. (C) After preprocessing, the TCGA cohort was used to train the CNN. CNN = convolutional neural network. (D) The trained CNN was evaluated on the independent validation cohort from our institution (in-house).

In our study, the CNN was trained to predict 5y-OS. The included WSIs were labelled as 5y-OS(**+**) or 5y-OS(**-**), depending on the survival time of the corresponding patient. The tumor region was annotated and patches were generated from the annotated tumor region. The patches retained the label that was defined for each WSI. The TCGA cohort served as the training set while the cohort from our institution was used for independent validation. The detailed pre-processing steps and the training of the model are described below and illustrated schematically in Fig 1.

### Pre-processing

Slides of eligible patients from TCGA were downloaded. For the validation cohort, a Leica Aperio AT2 DX was used to digitalize slides with 40-fold magnification, resulting in whole slide images (WSI) with a resolution of 0.25 μm/px. Tumor regions had been annotated by experienced pathologists (ZP, MS, TG). A grid of squares was used to tessellate the WSIs. Each square needs at least 50% overlap with the annotated region to qualify as a valid patch. Each patch was saved with a reference to the WSI it originated from (Fig 1A). Because different scanners were used to digitalize the WSIs all patches were downscaled to a uniform edge length of 129.38 μm x 129.38 μm per patch (0.2527 μm/px), resulting in 512px x 512px images. A patch was classified as blurry and therefore was discarded if the variation of the Laplacian was less than 170. To approximate the different degrees of coloration between WSIs, caused by H&E staining, the color space of all patches was stain-normalized using the Macenko method. A target image was needed as a template to indicate the direction in which to shift the color space (Fig 1B). WSIs containing less than 250 patches of ccRCC tissue of sufficient quality were excluded. After pre-processing the TCGA-KIRC cohort comprised 254 different patients with a total of 1,054,748 patches and our in-house cohort encompasses 99 patients with a total of 657,345 patches. WSIs were annotated and tessellated using QuPath version 0.2.3 [25]. Blur-detection was implemented in Python version 3.7.7 (Python Software Foundation, Beaverton, OR, USA).

### Model training

A ResNet18 CNN, pretrained on ImageNet, was trained to predict 5y-OS. The TCGA training set was divided into ten folds with similar distribution by stratifying with respect to outcome, metastasis, grading, and tumor size. Hyperparameters were tuned using a ten-fold cross-validation. The best CNN during cross-validation was trained in two stages. In the first stage, the fully connected layers (head) as well as the layers close to the input (body) were trained for ten epochs, using a learning rate of 1e-06. Subsequently, the whole model (head and body) was trained for additional 17 epochs in the second stage with a learning rate of 1e-07. Training followed Leslie Smith’s “one cycle policy” and learning rates were selected based on an algorithm that minimizes loss for a smaller sample of the training set while maximizing learning rate to speed up train time [26]. Patches were augmented according to Howard et al., using Flip, Warp, Rotate, Zoom, Brightness, and Contrast [27]. After hyperparameters were established, the model was trained using all 254 WSIs of the training set. Inference was carried out on all patches for each WSI of the independent test set. The CNN assigned a probability score for every patch. To determine the class for an entire WSI, the scores of all associated patches were averaged and classified as 5y-OS(**-**) (likely deceased) if the score exceeded a threshold of 0.5, otherwise as 5y-OS(**+**) (likely alive). Training as well as inference were implemented in Python 3.7.7, using PyTorch 1.6 [28] and fast.ai [27].

### Statistics

Area under the Receiver Operating Characteristic curve (AUROC) and balanced accuracy were used as metrics to evaluate the performance of the CNN algorithm. Standard deviations were reported for the cross-validation to show differences between splits and 95%-confidence intervals (95%-CI) were reported for the resulting model and its performance on the validation cohort. To calculate the 95%-CIs, the same model was trained ten times using the same data and established hyperparameters. The calculated probability scores were compared between both OS groups using a two-sided Mann-Whitney U test with a predefined significance level of p = 0.05. Furthermore, univariable Kaplan-Meier analysis including log-rank test and univariable Cox regression were performed using the non-binary survival time (in months). These calculations were conducted in Python 3.7.7 extended with the libraries SciPy and lifelines.

### Multivariable logistic regression models

Age, tumor stage (T1/T2 vs. T3/T4), grading (G1/G2 vs. G3/G4), sex (male vs. female) and metastasis status at surgery (M+ vs. M-) were used as clinicopathological variables for multivariable logistic regression analysis. A model including these variables was trained on the TCGA set using the statsmodel.api library in Python. Non-significant (p > 0.05) variables were dropped via backward elimination in the training process. The resulting model was evaluated on the training and validation cohort with and without the binary prediction of the best CNN.

## Results

### Patient population

**Fig 2:**
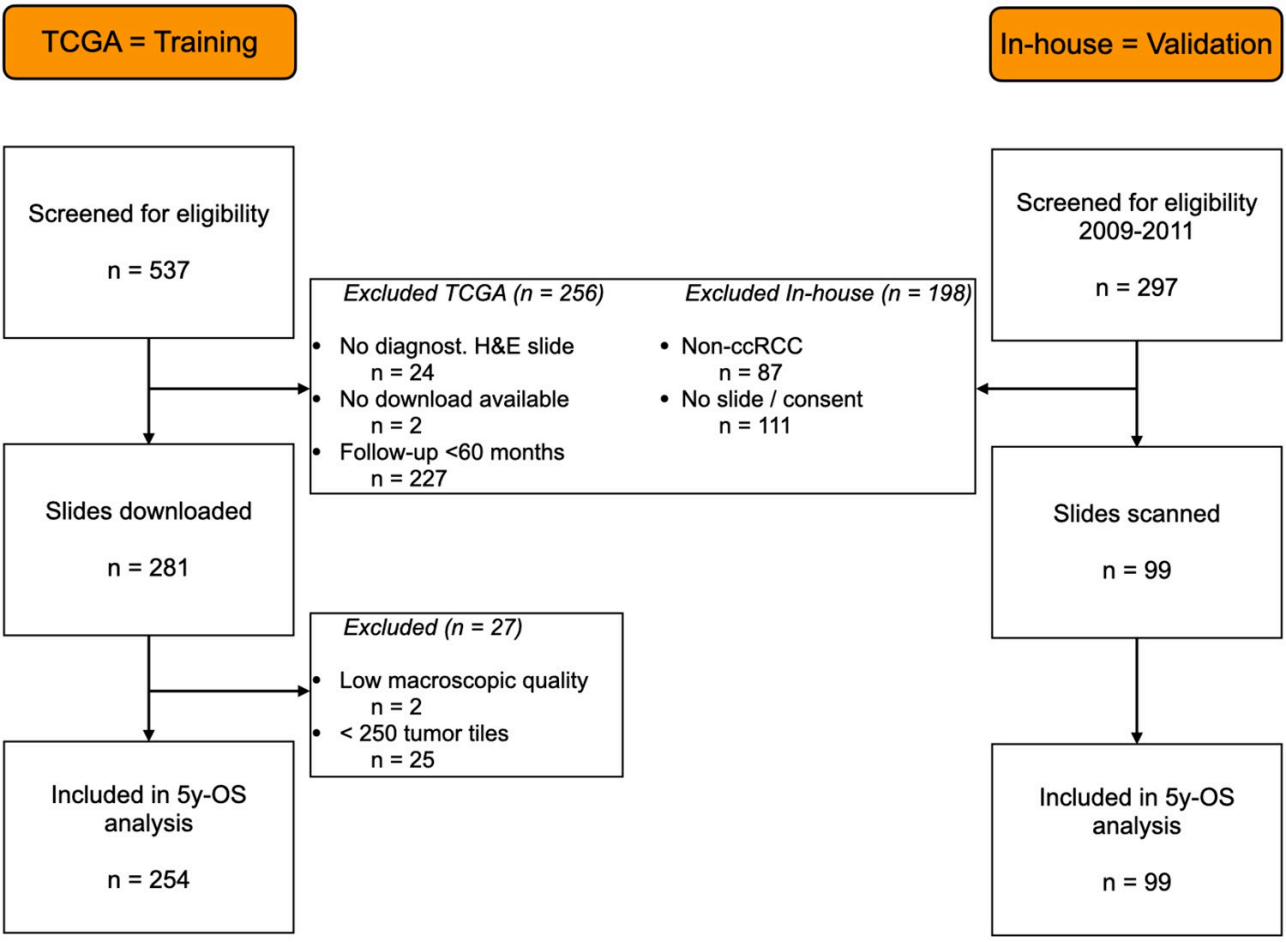
Flowchart of patient inclusion (one WSI per patient).

As depicted in Fig 2, 254 patients with corresponding WSIs from the TCGA screening cohort and 99 from the RCC cohort from our institution were included in the study.

The detailed patient characteristics are presented in Table 1. 53% (n = 134) of the patients had died < 5 years after diagnosis in the TCGA cohort and 14% (n = 14) in the validation cohort. A higher percentage of high grade ccRCCs (G3/G4) and metastases (M+) was seen in the TCGA cohort (n = 167, 65% and n = 66, 26%) compared to the validation cohort (n = 8, 8% and n = 10, 10%).

**Table 1:** Study population.

Variable	TCGA	In-house validation
**Patients (n)**	254	99
**Median age (years), IQR**	62 (53–72)	62 (53–69)
**Male, n (%)**	160 (63)	71 (72)
**Tumor size**		
** pT1, n (%)**	99 (39)	51 (52)
** pT2, n (%)**	39 (15)	13 (13)
** pT3, n (%)**	106 (42)	33 (33)
** pT4, n (%)**	10 (4)	2 (2)
**Grading**		
** G1, n (%)**	2 (1)	11 (11)
** G2, n (%)**	84 (33)	80 (81)
** G3, n (%)**	105 (41)	8 (8)
** G4, n (%)**	62 (24)	0 (0)
** GX, n (%)**	1 (0.3)	0 (0)
**Metastasis,**		
** M+, n (%)**	66 (26)	10 (10)
**Follow-up**		
** Median Follow-up, IQR (months)**	63 (21.5–81)	103 (90.75–116)
** Deaths during follow-up, n (%)**	155 (61)	25 (25)
**Deaths < 5 years, n (%)^a^**	134 (53)	13 (13)

^a^ used as label (5-year overall survival) for the CNN

G = grading; IQR = interquartile range; M = metastasis; n = number; OS = overall survival; TCGA = The Cancer Genome Atlas.

### Performance on the training and test set

**Fig 3:**
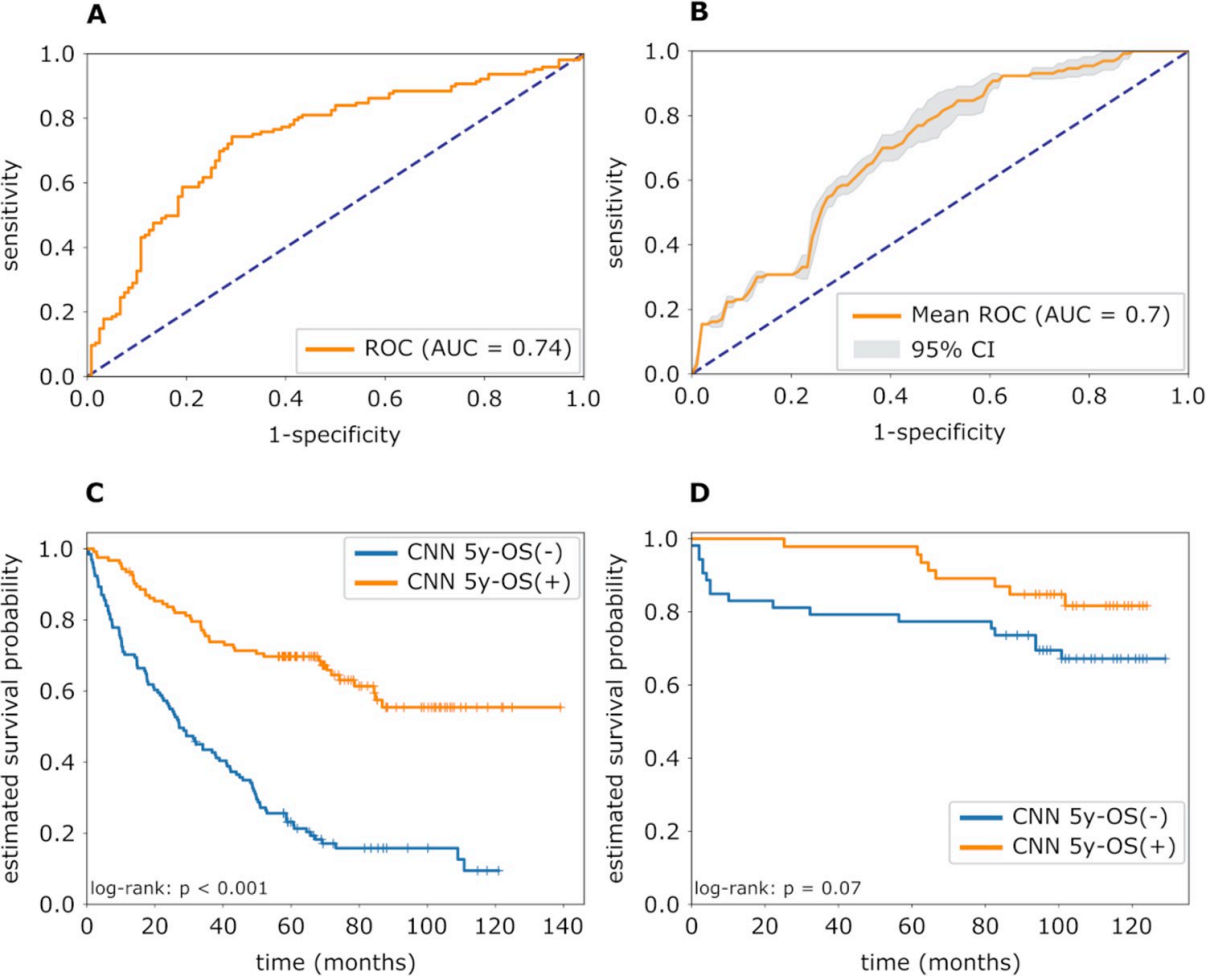
Prediction of overall survival in clear cell renal cell carcinoma using a CNN. (A) Mean ROC curve (orange) of the CNN’s prediction of 5y-OS on the training set. The dotted blue line represents the ROC curve resulting from random classification. The 1-specificity (false positive rate) was plotted on the x-axis and the sensitivity (true positive rate) on the y-axis. 5y-OS = 5-year overall survival; CNN = convolutional neural network; AUC = area under curve; ROC = receiver operating characteristics. (B) Mean ROC curve along with the 95% confidence interval (grey area) over ten identically trained CNNs on the validation cohort. (C) Kaplan-Meier curves grouped by the CNN-based classification and log-rank test for the training cohort. The blue curve shows the group predicted as 5y-OS(-) by the CNN, the orange curve shows the 5y-OS(+) group. (D) Kaplan-Meier curves and log-rank test for the validation cohort.

In the ten-fold cross-validation using the TCGA training set, a mean AUROC of 0.75 (standard deviation [SD] = 0.07), balanced accuracy of 72.0% (SD = 7.9%), sensitivity of 72.4% (SD = 10.6%) and specificity of 71.7% (SD = 11.9%) were achieved. On the validation cohort, the mean AUROC, balanced accuracy, sensitivity and specificity were 0.70 (95%-CI: 0.69–0.71), 65.5% (95%-CI: 62.9–68.1%), 86.2% (95%-CI: 81.8–90.5%) and 44.9% (95%-CI: 40.2–49.6%) respectively. AUROCs of the CNN’s performance on the TCGA training set and the validation cohort are shown in Fig 3A and 3B, respectively. For all model-runs on the test set, the Mann-Whitney U test showed significantly higher probability scores for slides of the 5y-OS(**-**) group (each p < 0.05).

The Kaplan-Meier curves for the training and test set are shown in Fig 3C and 3D. Survival analysis showed significant higher survival probabilities for the 5y-OS(**-**) group (log-rank: p < 0.001) on the training set as depicted in Fig 3C. Univariable Cox regression revealed a hazard ratio of 3.69 (95%-CI: 2.60–5.23, p < 0.001). On the validation cohort results did not quite achieve statistical significance (log-rank: p = 0.07; hazard ratio = 2.13, 95%-CI: 0.92–4.94, p = 0.08; Fig 3D).

### Combination of deep learning and clinicopathological prediction

In multivariable logistic regression analysis, age, tumor size and metastasis were significant predictors for 5y-OS while grading and sex were not in this cohort. The latter were removed in the backward elimination process. The resulting model showed an AUROC of 0.75 on the TCGA training set. Adding the CNN to this model further improved the AUROC to 0.81. The CNN’s prediction was an independent predictor (Odds ratio = 4.86, 95%-CI: 2.70–8.75, p < 0.001) in this model as depicted in Table 2. On the validation set, the clinical parameter model alone yielded an AUROC of 0.88, the same as the model including the CNN.

**Table 2:** Multivariable logistic regression for the prediction of 5-year overall survival trained on the TCGA cohort.

	ß	Odds Ratio	95%–CI	p-value
**Model containing clinical parameters only**				
Metastasis (M+ vs. M-)	1.29	3.63	1.47–7.56	0.001
Tumor size (T3/T4 vs. T1/T2)	1.13	3.09	1.72–5.53	< 0.001
Age (increase per 10 years)	0.26	1.31	1.04–1.64	0.02
**Model combining clinical parameters and CNN**				
CNN prediction (5y-OS(-) vs. 5y-OS(+))	1.58	4.86	2.70–8.75	< 0.001
Metastasis (M+ vs. M0)	1.01	2.74	1.26–5.96	0.01
Tumor size (T3/T4 vs. T1/T2)	0.99	2.69	1.43–5.05	0.002
Age (increase per 10 years)	0.18	1.19	0.93–1.52	0.16

5y-OS = 5-year overall survival; 95%-CI = 95% confidence interval; ß = Beta-coefficient; CNN = convolutional neural network; M = metastasis; TCGA = The Cancer Genome Atlas.

### Visual plausibility check of the CNN’s decision

**Fig 4:**
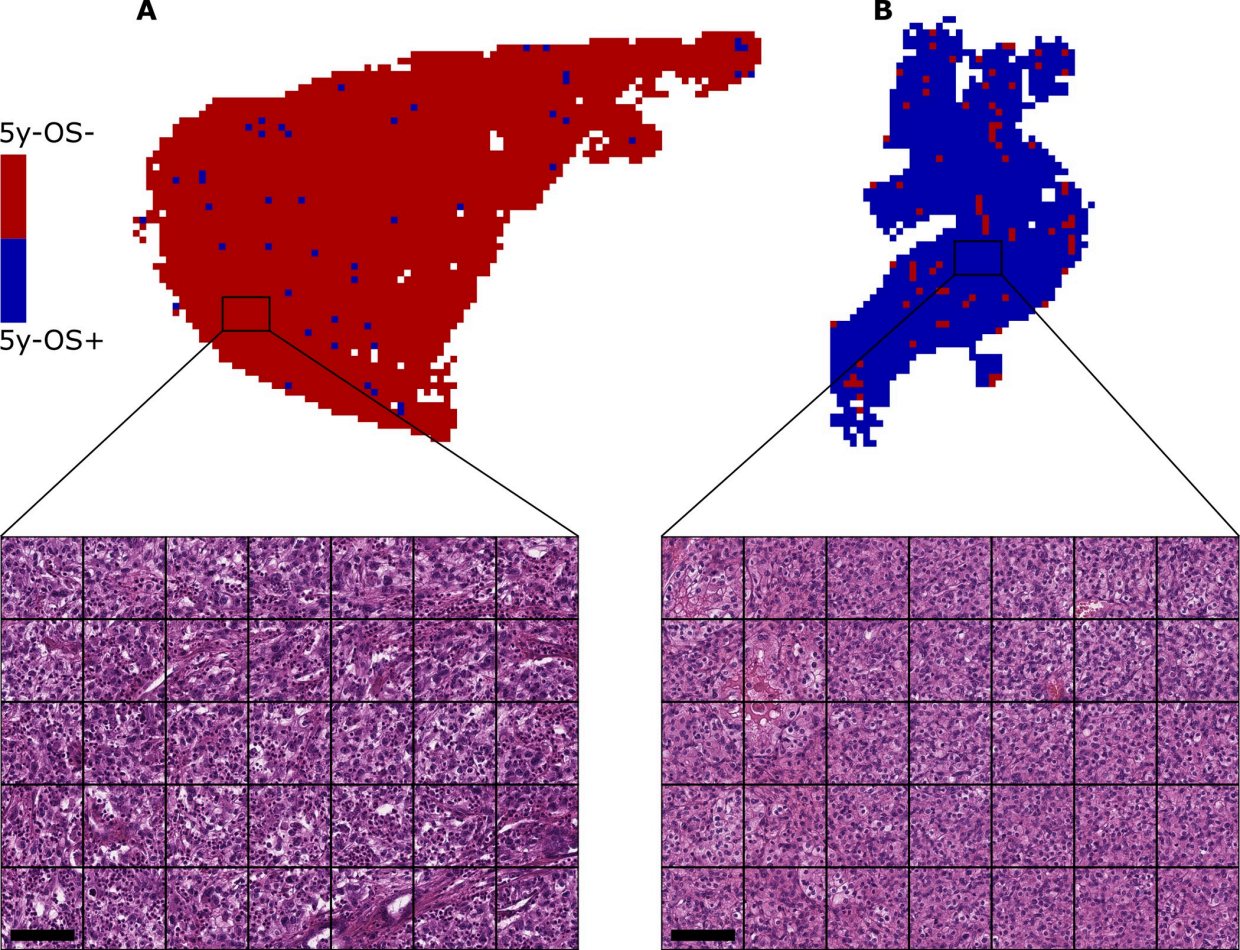
Exemplary prediction maps and corresponding H&E stain. Two prediction maps of slides that the CNN prognosticated correctly with high probability are shown. Blue patches indicate a probability score < 0.5 and thus classified as 5y-OS(+) while red patches indicate a score > 0.5 and thus classified as 5y-OS(-). 5y-OS = 5-year overall survival. CNN = convolutional neural network. (A) The slide correctly classified as 5y-OS(-) shows a high-grade renal cell carcinoma with greatly enlarged, partly multinuclear nucleoli, heterogeneous nuclear atypia and accompanying inflammatory reaction. Scale bar = 100μm. (B) The slide correctly classified as 5y-OS(+) shows a low-grade renal cell carcinoma with still mostly uniform nuclei and no areas of a more aggressive type. Scale bar = 100μm.

To better understand the CNN’s decision and to verify its general plausibility, slides that were correctly classified with high probability for 5y-OS(**-**) and 5y-OS(**+**) were evaluated as exemplarily shown in Fig 4. The upper part of the figure shows the CNN’s prediction of each patch of two ccRCC WSIs. Note that for such high-probability slides, a large majority of the individual patches is classified correctly. The lower part of the image shows a representative part of the corresponding histological image. Differences in the nuclear size, nuclear atypia and signs of inflammation are visible. The histopathological differences seen in these images, which are correctly classified by the CNN, thus allow the conclusion that the CNN makes plausible decisions in principle.

## Discussion

In the present study, a pretrained ResNet18 CNN was used to predict 5-year OS in ccRCC directly from H&E-stained diagnostic whole slide images. Good performances were seen in binary prediction on the training set. The results were validated on an independent external test set, demonstrating the generalizability of this method. Furthermore, the CNN-based classification was an independent predictor in a multivariable clinicopathological model.

Survival prediction is an ongoing challenge in RCC. Multiple different models have been developed already, but none of them has incorporated AI-based image analysis [29–31]. Thus, in our study, we trained a CNN to predict 5y-OS. On the TCGA cohort, a mean AUROC of 0.75 was achieved demonstrating that 5y-OS can be predicted directly from H&E-stained primary tumor slides in ccRCC. The external validation of our method on our in-house cohort, which is especially relevant if only data from one source is used for training [32], showed an AUROC of 0.70. Hence, the performance of our method showed only a moderate decline when transferred to an unseen cohort. This indicates that this method extracts generalizable tumor structures relevant for OS prediction from the H&E slides of ccRCC. Of note, AUROC is a well-established performance metric used to evaluate the CNNs ability to predict binary outcome since it is independent of the defined probability score threshold. The—threshold-dependent—sensitivity was higher in the validation cohort while specificity dropped. A change of the threshold, which we set at 0.5, would very likely optimize the sensitivity and specificity on the corresponding task [33]. However, due to the mostly exploratory nature of our study, a threshold optimization was not considered expedient at this stage.

In Cox regression analysis, groups based on the CNN’s classification of the slides in the TCGA cohort showed significant differences in survival with a hazard ratio of 3.69 (95%CI: 2.60–5.23, p<0.01) for the 5y-OS(**-**) group. Thus, although trained for the binary endpoint 5y-OS, this method shows the potential to be used in prognostication of continuous survival data. However, results did not quite reach statistical significance on the test set. The relatively small and imbalanced cohort with higher survival rates in comparison to the TCGA cohort might have contributed to this finding. Thus, a validation cohort including more slides and events may be needed to investigate the ability of the CNNs prediction to contribute to the prediction of overall survival more thoroughly.

The long-term goal of AI-based image medical analysis research is clinical implementation. The most important requirement is the clinical benefit of the technique. In other diagnostic tasks, near perfect accuracy comparable to that of pathologists was achieved, e.g. in automated tumor detection or grading of prostate cancer [34, 35]. In AI-based outcome prediction, the performance of the image-based analysis alone is expected to be worse due to the high number of different factors influencing oncological outcome. Therefore, we analyzed whether the combination of the CNN output and clinical parameters and known risk factors in a single prognostic model can provide an added benefit. The CNN result was an independent predictor in multivariable analysis in addition to metastasis, tumor size and age. Hence, the multivariable analyses demonstrated that the information provided by CNN-based analysis of histological slides is not redundant with the information provided by the other known risk factors, e.g. the occurrence of metastasis. Furthermore, the combination of the CNN and clinicopathological parameters yielded a higher AUROC on the TCGA training cohort than the model containing clinicopathological variables only. No improvement was seen on the test set. The main reason for this may be the accurate prediction of the clinicopathological model which yielded an AUROC of 0.88 as well as the limitations of the test set already discussed above.

Another important issue relevant for clinical implementation of AI-based systems in general is explainability or at least interpretability of the system. Due to its architecture, a CNN uses image features that are not defined beforehand. The definition and visualization of the relevant features remains a challenge and thus presents a hurdle in clinical implementation. We reanalyzed slides which were classified correctly with high probability / confidence by the CNN for each group. In re-pathological evaluation, patterns associated with aggressive tumor type / behaviour were seen on CNN-high-risk slides while the CNN-low-risk slides showed a non-aggressive / indolent morphology. Although such an evaluation cannot define the exact image features used by the CNN, it may serve as a concept check to demonstrate that the CNN’s decision is to some extent comparable to the traditional pathological evaluation of the tumor. This can help to interpret, check and entrust the CNN’s decision [36].

### Similar works

Our results are in line with other studies using the TCGA-KIRC cohort to identify prognostic biomarkers in ccRCC. Marostica et al. trained a CNN to differentiate between low and high-risk stage I RCC patients, which yielded a significant risk stratification in Cox-hazard analysis (log-rank test p = 0.02) [23]. Here, the focus of the study was on the development of a model for the detection of malignant cells as well as the differentiation of histological subgroups. In contrast to our study, the prediction of survival was investigated for stage I patients only. Tabibu et al. extracted predefined image features to predict OS using these features in a LASSO Cox model (hazard ratio = 2.26, p < 0.01) [21]. In this work, only high probability patches from a self-developed automated malignancy detection were used for prediction while in our study all tumor patches could be used for prediction. Survival prediction in other RCC subtypes also showed promising results. Cheng et al. developed a framework to use topological features to predict OS in papillary RCC patients [20]. An AUROC of 0.78 to predict 5-year OS was achieved. The main drawback in all mentioned studies was the lack of validation on an independent cohort. Although techniques, such as cross-validation, can help reduce the risk of overfitting the model to the training data, only external validation can truly demonstrate generalizability [32]. For models that have been trained with one cohort only, a moderate loss of performance is frequently observed on an independent validation set. This was also seen in our study. The use of larger and more diverse data sets is one of the crucial factors for the development of a generalizable model. Chen S et al. showed successful external validation for the prediction of disease-free survival using predefined image features in ccRCC patients (hazard ratio of machine learning risk score = 3.12, p = 0.034) [22]. Despite the different endpoint and methodology of this study as compared to our CNN-based study, the positive results of both studies demonstrate that there obviously is significant prognostic information encoded in the simple histological H&E images and that it can be extracted using computational image analysis. The results suggest that such methods may even lead to a better prognostication than is the case with the currently used histological classifications.

### Limitations

First, the retrospective nature of this study naturally represents a limitation. However, before prospective clinical trials can be conducted in the field of deep learning-based prognostication, algorithms need to be developed in such exploratory works. Second, a moderate performance drop was seen on the validation set, so overfitting may have been present to some degree. Generally, studies with larger consecutive cohorts are needed to reduce overfitting and to train and validate the CNN on the full morphologic spectrum of ccRCC including rare subtypes. The number of slides included in the training set is rather at the lower limit for training a ResNet CNN. By increasing the number of slides, a better and more stable performance might be achieved. Nonetheless, over one million patches were extracted from the TCGA slides and used for training, which can be considered enough, especially if the CNN is pretrained, as in our case. Third, since only patients with information on 5y-OS were included, there is risk of selection bias. Finally, a potential routine application requires digital histopathology, which might not yet be available in many institutions.

## Conclusion

CNN-based prognostication of overall survival using H&E-stained slides in ccRCC shows promising performance and generalizability and can be combined with existing clinicopathological parameters. This widely applicable technique shows the potential of artificial intelligence in image-based outcome prediction. Further research is needed to fine-tune this method and increase robustness. The inclusion of this method in existing risk stratification models or the development of new, combined models should be pursued in the future.

## Supporting information

S1 Data (XLSX)
